# Cantharadine Blistering Tissue, and Brown’s Tissue Dressing

**Published:** 1850-04

**Authors:** 


*Cantharidine blistering tissue, and Brown's tissue dressing.*—Mr. George D. Phelps, 46, Cliff street, New York, has sent to us samples of articles entitled as above, with which, in so far as examination goes, we are much prepossessed. The blistering tissue appears to be tissue paper, saturated with cantharidine, and is said to vesicate with as much certainty and more speedily, than the common blistering plaster. If so, it certainly is entitled to preference form its greater neatness, and convenience of application.

The tissue dressing is designed for blistered surfaces, burns, and superficial sores, and consists of tissue paper covered with a thin layer of unguent. Its advantages over other emollient dressings are, that it is more conveniently applied; is much lighter, in this particular being an artificial substitute for the epidermis; and does not become rancid by keeping.

Both articles come in sheets, twelve feet square, rolled, and nicely put up in tin boxes.

They seem to us to be real improvements, and we do not hesitate to recommend before trying them. The latter we shall do so soon as occasion offers.

